# Thermosensitive Progesterone Hydrogel: A Safe and Effective New Formulation for Vaginal Application

**DOI:** 10.1007/s11095-014-1616-8

**Published:** 2015-01-22

**Authors:** Aliyah Almomen, Sungpil Cho, Chieh-Hsiang Yang, Zhengzheng Li, Elke A. Jarboe, C. Matthew Peterson, Kang Moo Huh, Margit M. Janát-Amsbury

**Affiliations:** 1 Department of Obstetrics and Gynecology, Division of Gynecologic Oncology, University of Utah, Salt Lake City, Utah 84132 USA; 2 Department of Pharmaceutics and Pharmaceutical Chemistry, University of Utah, Salt Lake City, Utah 84112 USA; 3 Department of Bioengineering, University of Utah, Salt Lake City, Utah 84112 USA; 4 Department of Pathology, University of Utah, Salt Lake City, Utah 84112 USA; 5 Department of Polymer Science and Engineering, Chungnam National University, 220 Gung-dong, Yuseong-gu, Daejeon, 305-764 Republic of Korea; 6 School of Materials Science and Chemical Engineering, Tianjin University of Science & Technology, Tianjin, 300457 China

**Keywords:** glycol chitin, mucoadhesive, progesterone, thermosensitive, vaginal gel

## Abstract

**Purpose:**

The safe and functional delivery of progesterone through the vaginal route remains an unmet clinical need. The purpose of this work is to prepare a new progesterone (P4) gel for vaginal application using a thermosensitive mucoadhesive polymer, glycol chitin (GC).

**Method:**

Thermogelling, mucoadhesive, mechanical, and viscoelastic properties of GC and the new formulation were evaluated using rheometry. *In vitro* release profile and the bioactivity of P4 were determined using vaginal fluid simulant (VFS) pH 4.2, and PR-reporter gene assay, respectively. *In vitro* safety of the formulations was tested using (VK2/E6E7) vaginal epithelial cell line and *Lactobacillus Crispatus*. Finally, *in vivo* safety and the efficacy of this formulation were evaluated using an endometrial hypoplasia mouse model.

**Results:**

Results shows the aqueous solution of 5%; (w/v) GC loaded with 0.1%; (w/v) P4 prepared in pH 4.2, (GC-P4), forms a thermosensitive mucoadhesive hydrogel and can maintain stable physical properties at 37°C. GC-P4 gel release 50% of P4 in 4 h after exposure to VFS, and no significant decrease in % viability of VK2/E6E7 or *Lactobacillus* was found after exposure to 5% GC or GC-P4. GC-P4 does not exhibit obvious toxicities to vaginal tissue *in vivo* even after repeated application. Efficacy studies indicated that GC-P4 was capable of preventing the progression of simple endometrial hyperplasia (SEH) to complex atypical endometrial hyperplasia (CAEH) *in vivo*.

**Conclusions:**

Results indicates that GC-P4 retains many characteristics for an effective vaginal delivery system for P4. Therefore we believe that GC-P4 formulation is a promising alternative to current vaginal P4 formulation.

**Electronic supplementary material:**

The online version of this article (doi:10.1007/s11095-014-1616-8) contains supplementary material, which is available to authorized users.

## Introduction

Progesterone (P4) is a female steroidal hormone that plays a crucial role in maintaining normal physiology of the female reproductive system. P4 is mainly involved in benign, cycle-dependent tissue proliferation of the uterus, ovulation, implantation of the fertilized ovum, and maintenance of pregnancy (1). Other physiological actions of P4 include the proliferation of normal breast tissue, protection against bone loss, as well as regulation of sexual responsive signals in the brain (1). Based on its broad impact, the therapeutic use of P4 has been applied in the management of various female reproductive conditions such as the prevention of preterm birth, luteal phase support, and in hormonal replacement therapy (HRT) (2–4). Furthermore, the antiestrogenic effect of P4 on the endometrium led to the emergence of P4 therapy as a part of fertility–sparing treatment for endometrial hyperplasia (EH) and primary endometrial carcinoma (EC) (5).

Various different options of P4 administration and formulation are available. Oral administration of bio-identical P4 provides convenient dosing. However, poor bioavailability due to the hepatic first pass effect provides only low endometrial P4 concentrations (6). On the other hand, synthetic P4 known as progestins, are commonly associated with undesirable side effects ranging from acne and water retention to depression (7). To better facilitate the cellular absorption, intramuscular (IM) injections of P4 have become a popular administration route. Yet, pain with injection, irritation at the injection site, infections and possible abscesses leading to neuropathies in some cases, can reduce patient compliance, and therefore therapeutic efficacy (8). In light of the aforementioned limitations, the vaginal route has gained acceptance as a viable drug delivery route, especially in the management of obstetrical and gynecological conditions (9,10). In addition, the vaginal administration of P4 exhibits a preferential absorption and distribution to the uterus based on the uterine first pass effect, a direct transport mechanism for drugs from vagina to the uterus (11,12).

Although vaginal P4 formulations such as suppositories and creams show clear advantages over other routes including the avoidance of the hepatic first pass effect, ease of administration, and the provision of site-specific treatments, currently available P4 vaginal formulations exhibit major drawbacks (6). The challenging vaginal environment including body temperature (37°C), a more acidic pH (4.5–5.5), vaginal secretions, and the self-cleansing mechanisms lead to the leakage of almost all formulations resulting in significantly decreased vaginal residence times, local tissue irritation and inflammation, more frequent yeast infections, and most importantly the loss of active ingredients responsible for an unsatisfactory therapeutic effect (2,13).

Recently, thermosensitive polymers possessing a reversible sol–gel transition behavior in aqueous media have been utilized for vaginal delivery of various drugs such as 5-fluorouracil and metronidazole (9,10). Such gels were found to provide a convenient and effective treatment method, especially if combined with mucoadhesive agents (9). In a previous report, we introduced glycol chitin (GC), which has proven to be a suitable candidate for various biomedical applications including its use as an injectable drug delivery vehicle and a potential new material for tissue engineering purposes (14). In addition, chitosan amphiphilic derivatives are known to enhance the solubility of hydrophobic agents (15,16).

Based on the aforementioned characteristics, we report for the first time the application of GC in vaginal drug delivery and it usage as a carrier for P4. We hypothesized that GC-P4 provide a safe, effective, and a better alternative to currently available vaginal P4 formulations. In order to confirm our hypothesis, several rheological, release profiles, as well as *in vitro* and *in vivo* safety and efficacy studies of the hydrogel system were conducted.

## Materials and Methods

### Materials

Progesterone was purchased from Spectrum (Gardena, CA, USA). Glycol chitosan (DP ≥ 400), acetic anhydride (99.5%), and Porcine stomach derived Mucin type III were purchased from Sigma-Aldrich (St. Louis, MO, USA). Crinone® gel was purchased from Watson Pharmaceutics (Morristown, NJ, USA). Gynol II® was purchased from Revive (Madison, NJ, USA). All other chemical reagents used were of analytical grade and used without further purification.

### Cell Culture

VK2/E6E7, Human vaginal epitheliums cell line (CRL-2616) and 293 T human embryonic kidney cells line (CRL-3216) were purchased from the American Type Cell Culture collection (ATCC, Manassas, VA). VK2/E6E7 were cultured in Keratinocyte-Serum Free medium (Invitrogen, Grand Island, NY, USA), supplemented with 0.1 ng/ml human recombinant epidermal growth factor, 0.05 mg/ml bovine pituitary extract, 100 units/ml penicillin, 100 μg/ml streptomycin, and additional calcium chloride of 0.4 mM. 293 T were cultured in Dulbecco’s Modified Eagle’s Medium (DMEM) supplemented with 10% fetal bovine serum (FBS), 100 units/ ml penicillin, and 100 μg/ml streptomycin. Cells were grown and routinely maintained at 37°C in 10-cm^2^ culture dishes. *Lactobacillus crsipatus* was purchased from ATCC and grown in an ATCC medium 146 Lactobacillus MRS agar/broth (BD, Sparks, MD, USA) at 37°C.

### Animal Studies

Female Nu/Nu mice (6–8 week old) were obtained from Jackson Laboratory (Bar Harbor, ME USA). Mice were housed at the Center for Comparative Medicine Animal Facility and treated following approved Institutional Animal Care and Use Committee (IACUC) protocols and guidelines.

## Glycol Chitin (GC) Hydrogel for Vaginal Application

### Preparation of Glycol Chitin (GC)

GC with ≈90% degree of acetylation (DA) was chosen in this study and was synthesized and characterized as described previously (14). The synthetic method and results are described on Supporting Information and (Fig S1). GC solution was prepared according to the modified cold method (17). Briefly, GC was slowly added to cold citrate phosphate buffer (4°C, 0.1 M, pH 4.2) under gentle mixing and allowed to dissolve completely for 24 h at 4°C.

### Effect of Concentration and pH on GC Gelation

To test the effect of concentration and pH on GC gelation temperature (T_gel_), GC solutions were prepared at various concentrations of 5, 6, and 7%; (w/v) at pH 4.2. T_gel_ were determined by using a stress-controlled rheometer (AR 550; TA instrument, DE, USA). The rheometer was equipped with 20 mm 4° steel cone geometry. Frequency and oscillatory stress were maintained with constant values of 1 Hz and 5 Pa, respectively. The temperature sweep analyses were conducted in the range of 15–45°C with a heating rate of 1°C/min. The gelation point was considered to be at the crossover point between *G′* (elastic modulus) and *G″* (viscous modulus). Effect of pH on gelation was measured on the GC’s concentration showing a T_gel_ within the acceptable temperature range for mucosal application between (30–36°C) at pHs of 4.2, 6 or 7.2 (10).

### Rheological Examination of GC Mucoadhesive Characteristic

To determine mucoadhesive property of GC, a GC-mucin mixture was prepared at pH 4.2. The final concentrations of mucin and GC in the solutions were 4 and 5%; (w/v) respectively (18). GC and mucin were mixed prior to rheological evaluation. The linear viscoelastic region (LVR) for each sample was determined through a strain sweep measurement at a constant frequency of 1Hz and 37°C. Frequency sweep analysis was performed at a range of 0.1–10 Hz within the LVR of the gel (19). Mean *G′* for samples were extracted from frequency sweep spectra. Positive values of the absolute synergism parameter *(ΔG)*, which is a equal to the interaction between polymer and mucin, is an indicative of mucoadhesion (20). The following equation was used to calculate *ΔG* (20):

1 $$ \varDelta G={G}_{mix}^{\prime}\hbox{--} {G}_p^{\prime } $$

Where *G*_mix_^′^ and *G*_*p*_^′^ are the elastic moduli for polymer-mucin mixture and the polymer alone, respectively. The contribution of the mucin elastic modulus (*G*_*m*_^′^) was neglected in the equation since it was relatively low.

## Glycol Chitin-Progesterone (GC-P4) Hydrogel

### Solubility of P4 in GC Solutions

To test the solubility of P4 in GC, P4 was mixed in GC solutions (1–5%; w/v) and stirred for 24 h at 4°C. The clear GC-P4 solution collected from centrifugation (12,000 RPM, 4°C) was used to determine the amount of solubilized P4 employing reversed phase (RP)-HPLC (Agilent Chemstation for LC/MS system, CA, USA) equipped with a C18 column at 254 nm (21). A mixture of methanol/water (80:20; v/v) was used, as the mobile phase at a flow rate of 1 ml/min.

### Preparation of GC-P4 Hydrogel

GC-P4 gel was prepared by adding P4 into the 5% GC solution (pH 4.2) in a concentration that exceeded it solubility and stirred for 24 h at 4°C. The final P4 concentration in GC solution was of 0.1%; (w/v). Osmolarity of GC-P4 was 358.16 ± 6.5 mmol/kg. T_gel_ for GC-P4 hydrogel was assessed using the same method described earlier.

## Mechanical Characteristics of GC-P4 Hydrogel

### Dynamic Oscillatory Rheology

To determine the mechanical spectra of GC-P4 hydrogel, an oscillatory stress sweep step was conducted using a stressed-controlled rheometer equipped with 20 mm 4° steel cone geometry. One hundred and fifty microliter of GC-P4 was placed on the Palter plate. The sample was allowed to equilibrate for 5 min at room temperature 25°C as well as 37°C. An oscillatory stress step was conducted in the ranges of 0.5–1000 Pa at a frequency of 1 Hz. The critical oscillatory stress (COS), the maximum stress at which the linear value of *G′* is disturbed, was obtained from the stress step spectra (22). Mechanical spectra of Crinone® and GC-P4 were also determined to compare the strengths of the two gels in vaginal like environment. The value of stress representative of the viscoelastic region of GC-P4 was used in the oscillatory frequency analysis (5 Pa). Oscillatory frequency step was conducted in a frequency range of 0.1–10 Hz at 25 and 37°C (19,23).

### Steady Shear Viscosity (Flow rheology)

To determine flow behavior of GC-P4, a shear step was conducted in the biological relevant shear of 0.1–100 s^−1^, resembling shear exposed by passive seeping of vaginal epithelium all the way to shear during coitus, respectively (23–25). Frequency was kept constant at 1 Hz. The sample was allowed to equilibrate for 5 min at 25 and 37°C prior initiation of the shear step.

### Rate of GC-P4 Gelation

To determine the time required for GC-P4 to transform to a gel state at body temperature, a time sweep analysis was conducted. Time sweep analysis was set for 10 min at a constant frequency of 1 Hz in addition of 5 Pa oscillatory stress. The sample was set to equilibrate for 5 min at 15°C in the conditioning step to assure the liquid state of GC-P4 before initiating the time sweep. At the onset of time sweep, the temperature was raised to 37°C and gelation time was recorded.

## GC-P4 Hydrogel in Biologically Relevant Fluids

To determine rheological behavior of GC-P4 after dilution with fluids that usually presents in the vagina, viscosities and the effect of dilution on gelation were evaluated. Vaginal fluid simulant (VFS) of pH 4.2 and seminal fluid simulant (SFS) of pH 7.7 were prepared as described previously (26,27). GC-P4 hydrogels were diluted with VFS in a biologically relevant ratio of (1:4; VFS: GC-P4) and (1:6; VFS: GC-P4) (28). A fresh set of GC-P4 samples were prepared and diluted with SFS in a biologically relevant ratio of (1:1; SFS: GC-P4) (28). For the measurement of gel behavior following dilution, gel samples were left on the rheometer platform to equilibrate for 60 s prior to being mixed with either VFS or SFS. Steady shear viscosity step and time sweep analysis were conducted as described earlier.

## *In Vitro* Release of P4 from GC-P4 Hydrogel

### Swelling/Erosion of GC-P4 Hydrogel

To study the mechanisms of *in vitro* P4 release from the GC-P4, change in hydrogel weight as function of time following VFS exposure was measured. One ml of GC-P4 was placed into screw capped vials and incubated at 37°C. Following gel formation, 0.75 ml VFSs was placed over GC-P4. Vials were then placed on the platform of a MaxQ 4450 Benchtop Incubating Shaker (Thermoscientific, IA, USA). The incubation temperature was maintained at 37°C. Samples were subjected to a rotation speed of 70 rpm (providing shear rate of 50 s^−1^). At predetermined time intervals, VFSs were removed by pipette and replace by a fresh solvent. GC-P4 were weighed at each time intervals using a AG104 analytical balance (METTLE TOLEDO, Columbus, OH). The amounts of VFS removed at each time were used to further analyze the percentage of P4 released from the hydrogel.

### Determination of P4 Release from GC-P4 Hydrogel

To analyze the amount of P4 release from GC-P4 hydrogel, P4 was extracted from VFS using diethyl ether. Briefly, in a fume hood, 3 ml of diethyl ether were added to the VFSs. Samples were vortexed for 1 minutest at room temperature and then placed in −20°C until complete freezing of the aqueous phase occurred. The organic layer was then removed and the samples were kept in a fume hood until complete evaporation of the organic phase. Samples were then suspended in 80 v/v% ethanol and the amount of P4 was determined (21). Quantitative determination of P4 was conducted using a linear calibration curve between the range of 1.96–62.5 μg/ml and a 0.99 correlation coefficient.

### Reporter Gene Assay

To confirm the bioactivity of P4 released from GC-P4 system, the ability of P4 to induce Progesterone receptor (PR)-signal transduction pathways was performed. Briefly, 239 T cells were seeded in a density of 1.5 × 10^6^ cells / well in a 6-well plate. A plasmid containing P4 responsive elements (PRE) fused to firefly luciferase (Cignal reporter assay kit, QIAGEN, CA, USA) was transfected into 239 T (500 ng /well). Transfection was carried out using FuGENE® 6 Transfection Reagent (Promega, WI, USA). 24 h post-transfection, cells were treated with either P4_100nm_, GC-P4_100nm_, GC, or no treatment for 18 h. Luciferase activity was measured using Dual-Luciferase Reporter Assay System (Promega, WI, USA) according to manufacture’s instruction. Results were represented as Luciferase activity normalized to Renilla luciferase.

## *In Vitro* Safety and Toxicity of GC-P4 Hydrogel

### MTT 3-(4,5-dimethylthiazol-2-yl)-2,5-diphenyltetrazolium Bromide Assay

To determine GC biocompatibility with vaginal epithelium, VK2/E6E7 cells were seeded in 96-well plate at a density of 5 × 10^3^ cell/well and incubated for 24 h in a volume of 100 μl media/well. Serial dilutions of GC in citrate-phosphate buffer (0.1 M, pH 4.2) were added to the cells and incubated for another 24 h. After exposure, 20 μl of 2.5 mg/ml MTT (Invitrogen, OR, USA) in PBS was added to the cells. The cells were incubated with MTT for 4 h 37°C. MTT solutions were completely removed and 100 μl DMSO was added to solubilize formazan crystals. Absorbance was measured at 540 nm using Spectramax 250 microplate reader (Molecular device, CA, USA). Percent viability was calculated from (OD_540_ sample / OD_540_ control) × 100, referring to GC treated and non-treated cells.

### Lactobacillus Viability Assay

To assess the effect of GC on the sustainability of an intact, normal vaginal flora, the viability of *Lactobacillus crispatus* after the exposure to GC was determined. Bacteria were seeded in a density of 10^7^ colony formation units (CFU) / 100 μl in 96-well plates and incubated with 100 μl serial dilutions of GC at 37°C for 24 h. After 24 h incubation, a 20 μl MTS [3-(4,5-dimethyl thiazole-2-yl)-5-(3-carboxymethoxyphenyl)-2-(4-sulfonyl) 2H-tetrazolium (Promega, Madison, WI, USA) reagent was added to each well. Absorbance was measured at 490 nm and the percent viability was calculated from (OD_490_ sample/ OD_490_ control) × 100, which refers to GC treated and non-treated bacteria. To confirm the safety of GC in the concentrating used for the GC-P4 formulation, the ability of 5% GC as well GC-P4 to inhibit the growth of *Lactobacillus* on an agar plate was tested. A scrap of *Lactobacillus crispatus* from bacterial broth was directly spread on an MRS agar plate. Each plate was divided into four quarters and 250 ul of the tested material was placed in the center of each quarter. Plates were incubated for 24 h at 37°C. The effect of 5% GC and GC-P4 on bacterial growth were evaluated and compared to the effect of both Gynol II® (nonoxynol-9) and Crinone®.

### Clonogenic Assay

Human VK2/E6E7 were seeded in six well plates (Transwell®, Costar, NY, USA) in a density of 2000 cells/ well. Cells were incubated in 2 ml Keratinocyte-Serum Free medium until complete cells attachment. Cells were then treated with either 5% GC, GC-P4_200μg_, P4_200μg_, Gynol II® as a positive control, or Crinone®_200μg_, through a 0.4 μm polyester transwell membrane for 24 h. In addition, one group of cells was left untreated and served as a blank. Transwells were then removed and cells were cultured for additional 7 days until the formation of visible colonies. Colonies were then fixed for at least 30 min with a 6% glutaraldehyde and 0.5 crystal violets mixture. Wells were rinsed under running tap water and numbers of blue visual colonies were quantified with Image J 1.44o software (N.I.H., Bethesda, MD, USA) (size range of 2~200 μm).

## *In Vivo* Safety and Efficacy of GC-P4 Hydrogel

### *In Vivo* Safety of GC-P4 Gel to Vaginal Epithelium

To investigate the safety of GC-P4 gel when applied to mouse vaginal epithelium *in vivo*, mice were divided into three groups (*n* = 2), anesthetized by IP injection with Xylazine-Ketamine at dose of 0.1 ml / 10 g and treated vaginally with either GC-P4 gel, Gynol II® (positive control), or phosphate buffered saline (PBS) (negative control) once a day for three consecutive days. After 24 h of administering the last dose, all animals were sacrificed and vaginal tissues were trimmed and fixed in 10% formalin for 48 h. Tissues were dehydrated in 70% ethanol and send to Associated Regional and University Pathologists (ARUP) at the University of Utah for Hematoxylin and Eosin staining (H&E). Histology of vaginal tissues for both treated and control animals were examined by two independent pathologists, using light microscopy at the department of Pathology, University of Utah.

### *In Vivo* Efficacy of GC-P4 Gel in Estrogen (E2) Induced Endometrial Hyperplasia (EH) Mouse Model

6–8 week old female Nu/Nu mice were divided in to three groups and anesthetized. Doses of subcutaneous estrogen (E2) were administered to mice in all groups to induce EH. One group of mice did not receive E2 therapy and served as disease negative control. After 4 weeks of E2 treatment and confirmation of simple endometrial hyperplasia (SEH) through histology, one group of animals, which received E2 therapy, received daily intravaginal GC-P4 (60 μg/day) treatment for 2 weeks by restraining the mice in rodent restrainer. The second group of E2 treated mice, received no P4 treatment and served as disease positive control. In 24 h of administering the last GC-P4 dose, animals were sacrificed and uterine tissues were trimmed, handled as mentioned earlier, and send to ARUP for H&E. Histology of GC-P4 treated and non-treated mice uteri were examined by two independent pathologists using light microscopy at the department of Pathology, University of Utah.

## Statistical Analysis

Statistical analysis and plotting graphs were performed using GraphPad Prism software (GraphPad Software Inc. San Diego, CA). All experiments are conducted in triplicate unless indicated otherwise. Results are expressed as mean ± SEM, and *P* < 0.05 was considered significant.

## Results

### Glycol Chitin (GC) Hydrogel for Vaginal Application

**Fig. 1:**
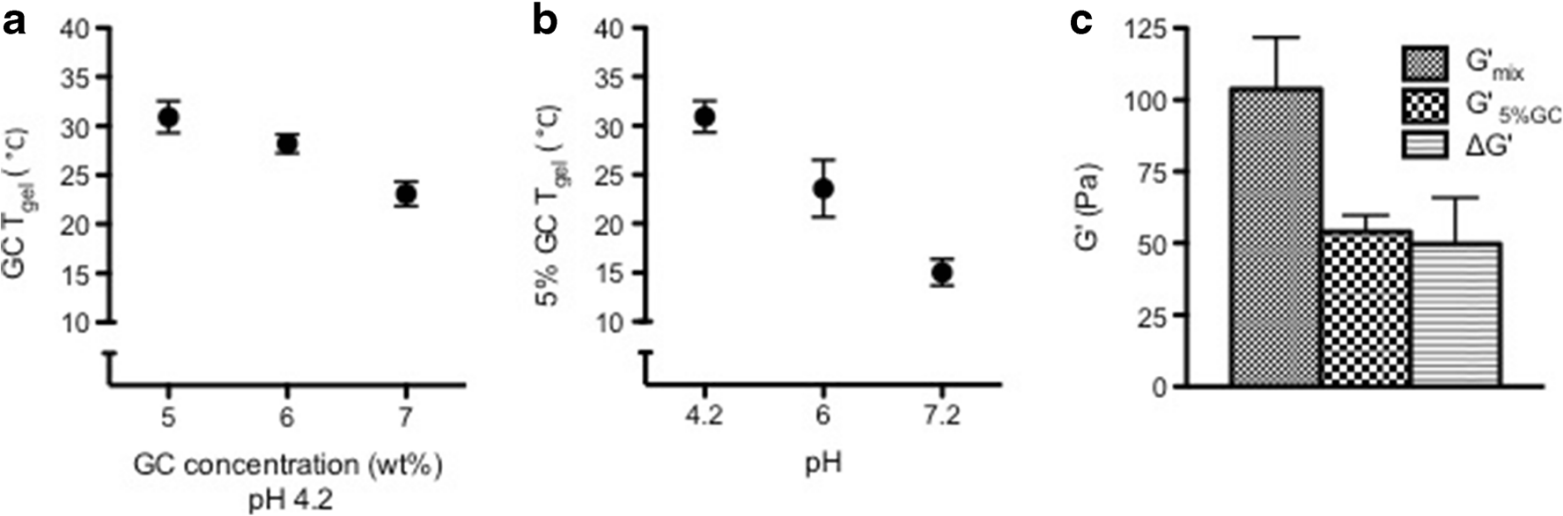
Glycol chitin (GC) hydrogel for vaginal application. (**a**) GC T_gel_, at pH 4.2 decreased by increasing polymer concentration, and (**b**) environmental pH in case of 5 wt % GC. (**c**) 5% GC retained mucoadhesive properties detected by a positive value of the interaction parameter *ΔG* and a *G*_*mix*_^′^ > *G*_*GC*_^′^.

Although vaginal P4 administration has demonstrated promising potency for many obstetrical and gynecological conditions when compared to other routes (oral and IM), environmental limitations such as acidic pH and the abundance of mucin in the vagina are challenging when aiming at the broader clinical translation of vaginal drug delivery. To confirm that GC hydrogel is capable to successfully overcome these challenges, we began the characterization by first measuring the 90% DA GCs T_gel_ as a function of concentration or pH. Ninety percent DA GC was chosen in this study based on its ability to form a low viscosity solution in room temperature, which easy to handle and to corporate drug into, and having a T_gel_ near body temperature using minimum polymer concentration (14). Other DA GC, *i.e.* 80%, was tested in the polymer selection process, however showed either no gelation in low concentration, or give high T_gel_ (>40°C) in higher polymer concentration (Figure S2). T_gel_ for GC was found to decrease with increasing GC concentration, indicating 30.9 ± 1.6°C, 28.2 ± 1°C, and 23.1 ± 1.3°C, at 5, 6, & and 7%, respectively (Fig. 1a, Figure S3). In addition, the T_gel_ at 5% demonstrated 30.9 ± 1.6, 26.5 ± 2.9, and 15 ± 1.3°C at a pH of 4.2, 6, and 7.2, respectively (Fig. 1b, Figure S4). Rheological analysis of GC demonstrated a positive rheological synergism parameter (*ΔG* > 0) that existed between GC and mucin under physiological pH (~4.2) indicating its mucoadhesive capability as shown in Fig. 1c.

### Glycol Chitin-Progesterone (GC-P4) Hydrogel

**Fig. 2:**
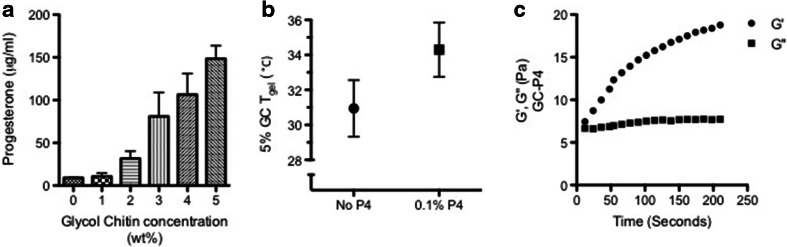
Glycol chitin-Progesterone (GC-P4) hydrogel. (**a**) Solubility of P4 in GC solutions. P4 solubility proportionally increased with increasing GC concentration, (*n* = 4). (**b**) 0.1%, (w/v) P4 loaded into 5% GC at pH 4.2 (GC-P4) maintains a T_gel_ near body temperature. (**c**) Gelation of GC-P4 within seconds after exposure to vaginal temperature (37°C).

Water-soluble amphiphilic polymers have been frequently studied for solubilization of hydrophobic drugs. Therefore, we tested the GC solubilizing capacity of P4. P4 solubility was found to proportionally increase with increasing GC concentrations reaching 148.4 ± 13.8 μg/ml in 5% GC (Fig. 2a). This did not exceed the solubility of P4 when using other formulations such as a mixture of PEG-400/water (50:50 v/v) (29). To achieve therapeutically relevant amounts, we further increased the amount of P4 loaded within the gel, and P4 was added to 5% GC solution in a concentration that exceeded its solubility. Five percent GC solution containing P4 0.1%; (w/v) at pH 4.2, which will be referred to as GC-P4 in the rest of this manuscript, demonstrated a slight increase in T_gel_ from 30.9 ± 1.6°C to 34.3 ± 1.6°C (Fig. 2b), which was statistically non-significant (NS). Moreover, GC-P4 formed a stable hydrogel in several seconds within vaginal (body) temperature (Fig. 2c).

### Mechanical Characteristics of GC-P4 Hydrogel

**Fig. 3:**
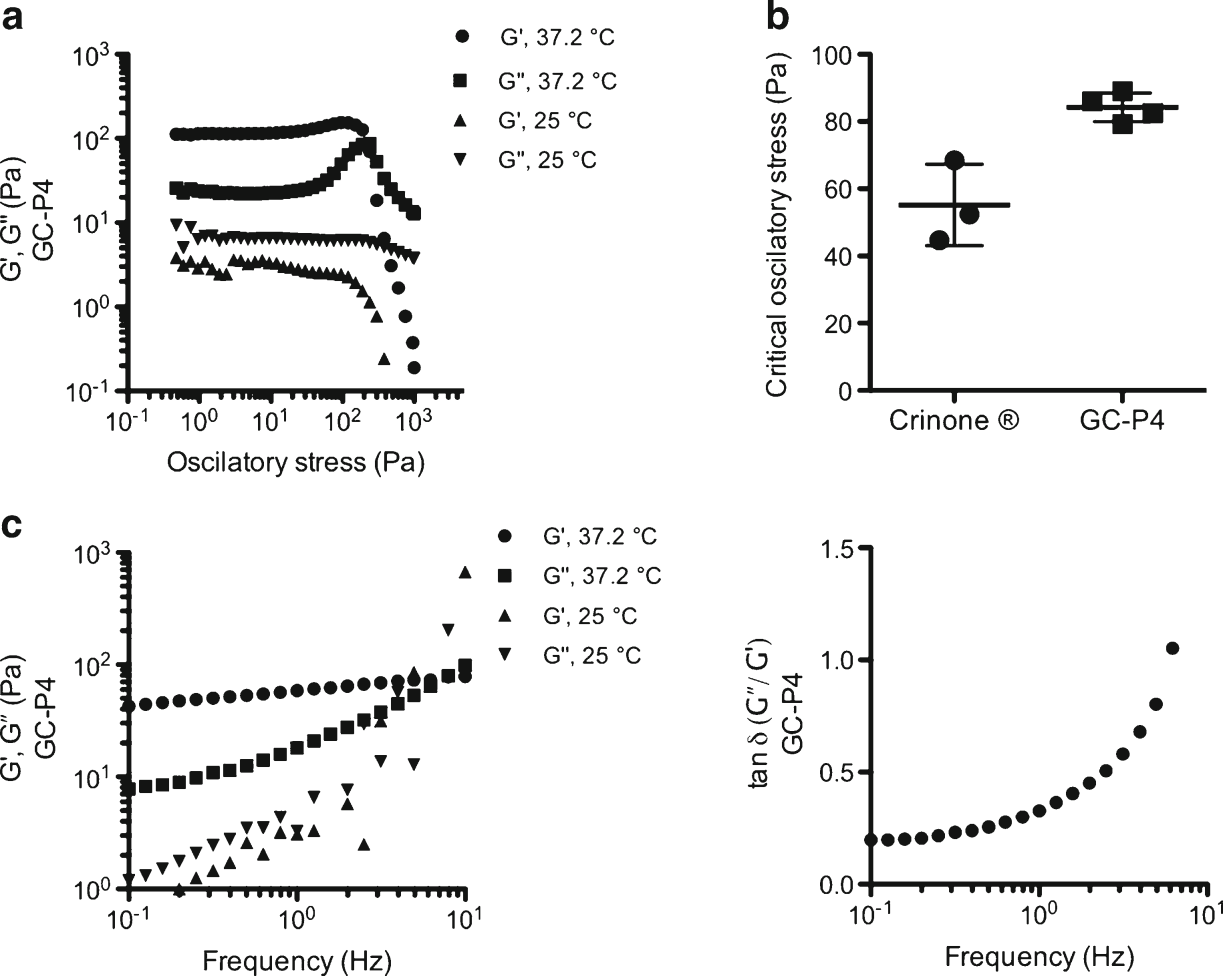
Mechanical characteristics of GC-P4 hydrogel. (**a**) Stress sweep analysis for GC-P4 gel at 25 and 37°C. (**b**) COS_GC-p4_ > COS_Crinone®_ indicating that GC-P4 gel is more resistance to external stress, at 37°C. (**c**) Frequency spectra in 37°C indicated that GC-P4 gel exhibited elastic properties in vaginal environment, unlike in 25°C. (**d**) tan δ = (*G″/ G′*) < 1 for most frequency range for GC-P4 in 37°C typical for elastic-solid materials.

Confirming suitable mechanical characteristics equipping our material with necessary properties to withstand the vaginal environment, we studied the dynamics of GC-P4 rheological properties in 25 and 37°C. Unlike at 25°C, GC-P4 revealed a solid-like behavior with a rigid three dimensional microstructure under oscillatory stress ranging from 0.5 to 100 Pa in 37°C (Fig. 3a). When measuring critical oscillatory stress (COS), GC-P4 formed a 1.5 times firmer gel than Crinone® as shown in Fig. 3b. In addition, GC-P4 had a dominant elastic property (*G′* > *G″*) during frequency sweep analyses at 37°C (Fig. 3c) and indicated a tan δ = *G″ / G′* < 1 as shown in Fig. 3d. Where at 25°C, GC-P4 showed a typical liquid material frequency spectra of *G′* < *G″*, and frequency dependent viscoelastic moduli.

### GC-P4 Hydrogel in Biologically Relevant Fluids

**Fig. 4:**
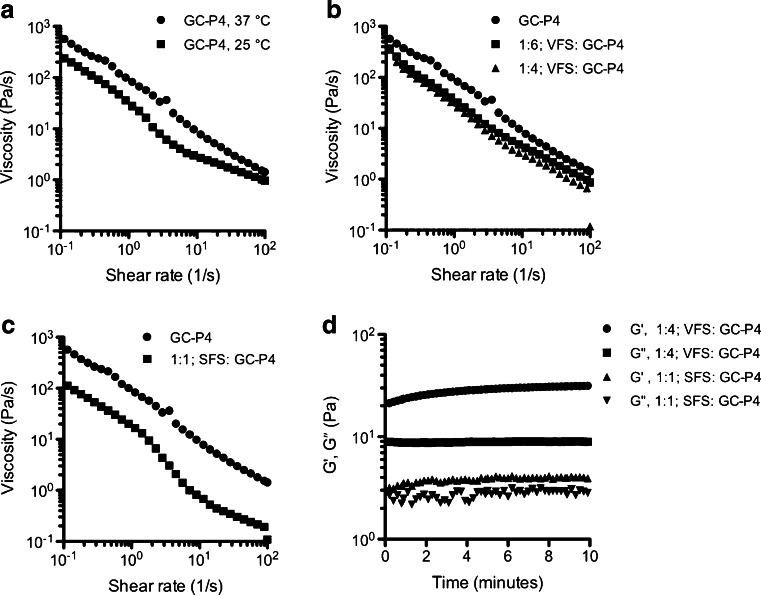
GC-P4 hydrogel in biologically relevant fluids. (**a**) GC-P4 presented a non-Newtonian viscosity (shear thinning) in shear rate of 0.1–100 s^−1^ 37°C, and a significant reduction of GC viscosity at 25°C. (**b**) GC-P4 viscosity non-significantly decreased after 1:6 dilution with VFS and significantly decreased after 1:4 dilution. (**c**) Dilution with SFS had a greater impact on GC-P4 viscosity. (**d**) Representative figures of GC-P4 time sweep analyses after dilution with biological relevant fluids (*n* = 2). No effect on gel quality occurred after 1:4 dilution of GC-P4 with VFS, however, a decrease in gel quality was found after 1:1 dilution with SFS detected by unstable and closer *G′* and *G″* values.

Viscosity profiles showed that GC-P4 hydrogel presented a pseudoplastic non-Newtonian behavior (shear thinning) with a biological relevant shear rate at 37°C (Fig. 4a). A significant reduction in the viscosity of GC-P4 occurred at 25°C. Under the influence of relevant biological fluids, GC-P4 showed a slight decrease in viscosities under dilution with VFS (Fig. 4a). However, as expected the viscosity of GC-P4 was significantly decreased after the dilution with a SFS (Fig. 4b). Time sweep analyses also showed destabilized viscoelastic moduli of GC-P4 and a reduction in the magnitude between *G′* and *G″* when diluted with SFS (Fig. 4d). Mimicking prevalent physiological conditions, GC-P4 diluted with VFS clearly yielded a stable gel with constant viscoelastic moduli (Fig. 4c). GC-P4 was capable of maintaining this stable gel state over 12 h showed in (Figure S5)

### *In Vitro* Release of P4 from GC-P4 Hydrogel

**Fig. 5:**
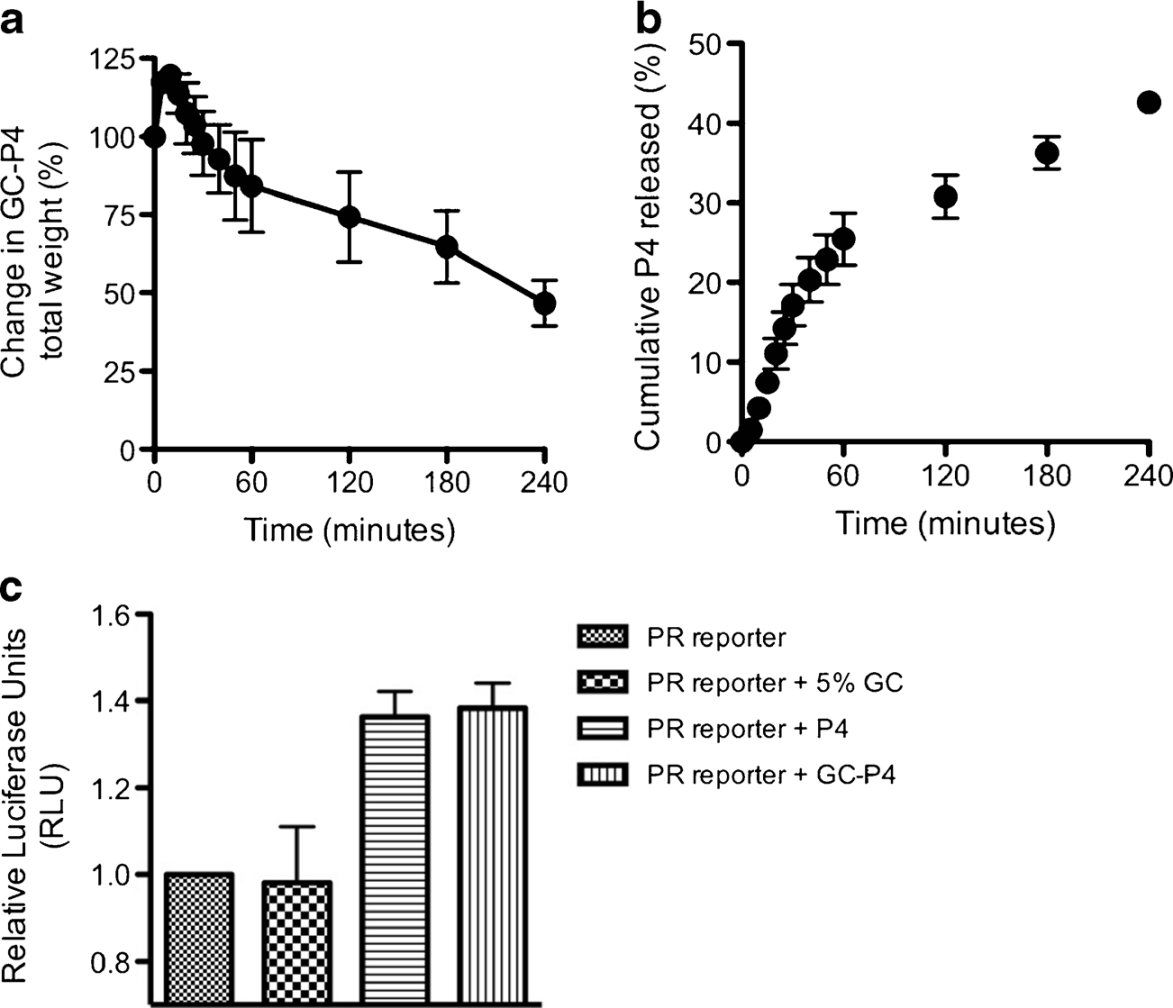
*In vitro* release profile and bioactivity of P4 released from GC-P4 hydrogel. (**a**) Swelling of GC-P4 gel occurred within the first 30 min after gel exposure to VFS at 37°C, followed by decreased in weight (erosion) (*n* = 4). (**b**) Up to 50% of P4 dose was released within 4 h of exposure to VFS at 37°C (*n* = 4). (**c**) Increase in PR transcriptional activity after treatment with GC-P4_100nM_ measured in RLU with no significance different to RLU of P4_100nM_ treated cells.

To determine the mechanisms of P4 release from GC-P4 and estimate the gel durability, the hydrogel was exposed to VFS at 37°C, and a shear of 50 s^−1^ (30). GC-P4 exhibited a 20% increase in weight, which occurred within the first 30 min of exposure, followed by a weight decrease. Weight decrease reached up to 50% of GC-P4’s original weight following four hours of exposure to VFS (Fig. 5a). Consequently, 50% of P4 was released from the hydrogel within the same amount of time (Fig. 5b).

In addition we also investigated the bioactivity of the released P4 by progesterone receptor (PR) reporter assay capturing only active P4 binding to PR. P4 released from GC-P4_100nM_ showed comparable activity with free P4_100nM_ and also demonstrated significant increase in PR reporter activity compared to P4 non-treated and 5% GC only treated cells (Fig. 5c).

### *In Vitro* Safety and Toxicity of GC-P4 Hydrogel

**Fig. 6:**
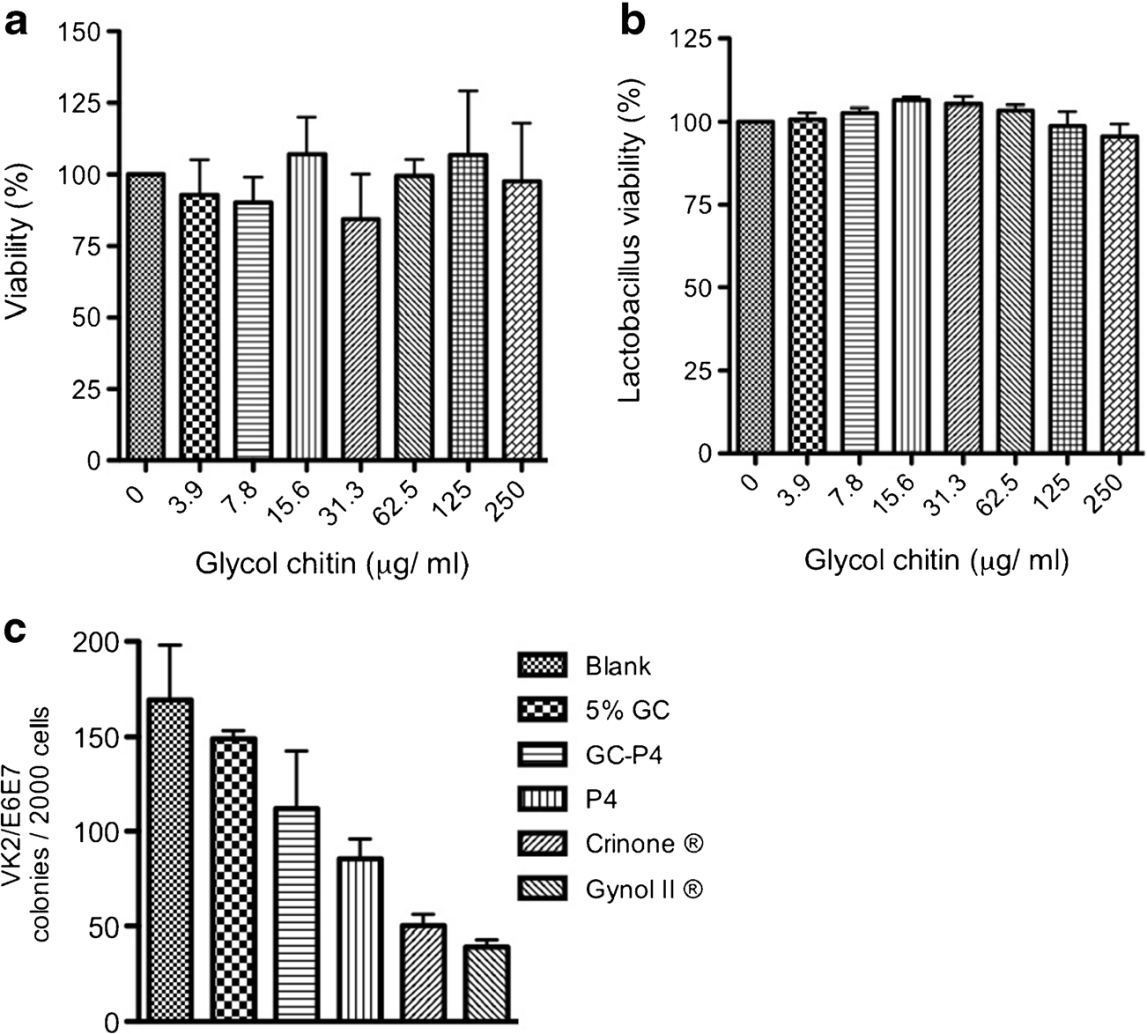
*In vitro* safety and toxicity of GC-P4 hydrogel. (**a**) MTT assays indicated no difference in percent viability in VK2/E6E7 cell after treatment with GC solutions. (**b**) No difference between % viability of *Lactobacillus crispatus* after treatment with different concentration of GC compared to non-treated cells. (**c**) VK2/E6E7 cell lines retained their ability to form colonies after treatment with 5% GC and GC-P4_200μg_ gel compared to P4_200μg_ alone, Gynol II®, and Crinone®_200μg_.

To assess the safety and toxicity of GC-P4 as a vaginal drug delivery system, cellular and microbial toxicity of GC and GC-P4 to human vaginal epithelial cells as well as normal, physiological vaginal flora were measured. MTT assays showed no toxicity towards the human vaginal epithelial cell line (VK2/E6E7) following exposure to different GC concentrations, indicated by (%) viability ranging from 84.4 ± 15.8 to 107 ± 22.3 (NS) when compared to untreated control cells (Fig. 6a).

*Lactobacilli* act as gatekeepers for the vaginal ecosystem and play a crucial role in protecting the vaginal environment from pathogens. MTS assays were performed to evaluate lactobacillus viability following exposure to various GC concentrations. GC did not impact viability of *Lactobacilli* showing (%) viability ranging from 95.6 ± 3.7 to 106.4 ± 1 (NS) (Fig. 6b). Further, there was no effect on *Lactobacillus* growth on the agar plate after exposure to 5% GC or GC-P4. However, Crinone® and Gynol II® inhibited the growth of bacteria on the agar plate (Figure S6).

In addition, clonogenic assays demonstrated that the number of VK2/E6E7 cell colonies following exposures to 5% GC was comparable to number of colonies in the non-treated group. Although there was a slight decrease in the number of VK2/E6E7 colonies treated with GC-P4, the formation of colonies were significantly reduced after treatment with Crinone® or Gynol II® (Fig. 6c, Figure S7).

### *In Vivo* Safety and Efficacy of GC-P4 Hydrogel

**Fig. 7:**
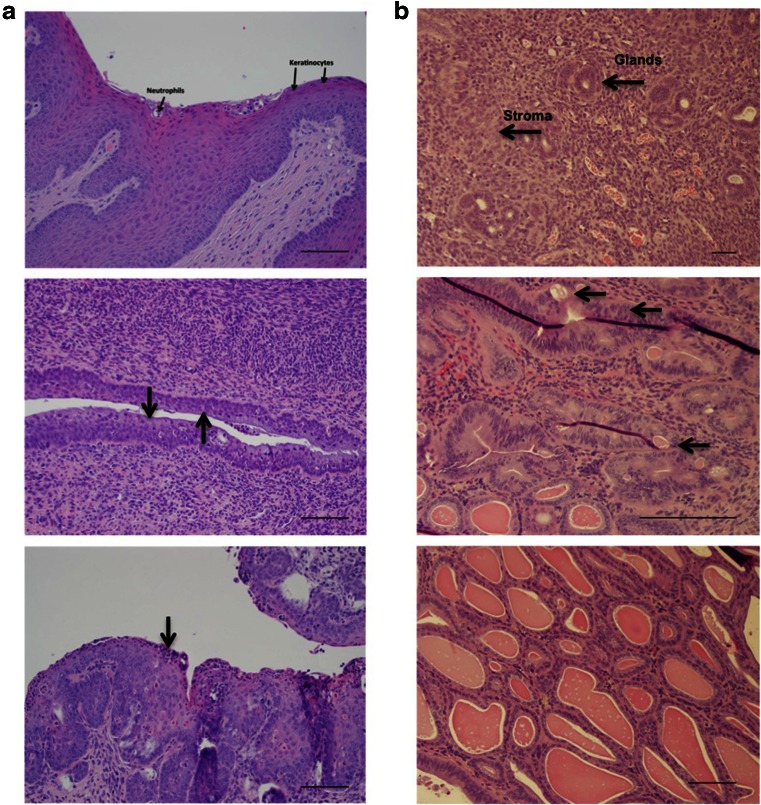
*In vivo* safety and efficacy of GC-P4 hydrogel. (**a**) Hematoxylin and Eosin staining (H&E) PBS (*Top*) with Intact squamous cells (Keratinocytes) and rare Neutrophil cells, Gynol II® gel (*Middle*) *arrows* indicate massive cell death of squamous cells (Keratinocytes), and GC-P4 (*bottom*) *arrow* indicate minimal cell death of Keratinocytes, X200. (**b**) Normal, murine endometrium demonstrates small tubular glands, some in clusters, but overall with abundant intervening stroma X100 (*top*). E2 Effect, no GC-P4 treatment X400 (*middle*) demonstrating complex atypical hyperplasia. Following initial daily treatment with vaginal GC-P4_60μg_ for 2 weeks, the features are consistent with simple non-atypical hyperplasia X200 (*bottom*). Scale bar = 100um.

To evaluate the safety and efficacy of GC-P4 following vaginal administration *in vivo*, GC-P4 was evaluated in a mouse model. Six to eight week old female Nu/Nu mice were repeatedly treated by vaginal administration of either GC-P4, Gynol II® (positive control), or PBS (negative control). Histopathological tissue evaluation was determinedas study endpoint. Within 24 h following the last dose, animals were sacrificed, tissues were harvested and vaginal epithelium underwent H&E staining followed by independent histopathological evaluation through two different anatomical pathologists. GC-P4 was determined to be safe to administer to murine vaginal epithelium *in vivo*. Only negligible, non-significant, transient signs of acute inflammation were observed when compared to a control group treated with PBS (Fig. 7a top and bottom). In contrast, treatment with Gynol II® showed ubiquitous signs of chronic vaginal tissue inflammation accompanied by massive death of squamous cells as well as a significant increase in neutrophils were observed (Fig. 7a middle).

To further determine the efficacy of GC-P4 *in vivo*, we designed and developed an E2 induced EH mouse model in which mice were exposed to a continuous dose of exogenous E2 over time. Female 6–8 week old Nu/Nu mice were assigned randomly to receive no E2 or GC-P4_60μg_ (normal endometrium), subcutaneous (s.c.) E2 only (disease control), or s.c. E2 followed by GC-P4_60μg_ (treatment group). GC-P4_60μg_ was administered vaginally once daily for 2 weeks after confirming the successful development SEH. Within 24 h after receiving the last dose mice were sacrificed. Endometrial tissues were harvested and underwent H&E staining followed by histopathological evaluation. As expected, normal, untreated mouse endometrium showed small tubular glands, some in clusters, but overall with abundant intervening stroma representative of healthy endometrium. Occasional mitotic figures were noted in endometrial glandular cells, consistent with proliferative phase endometrium (Fig. 7b top). Endometrial samples derived from mice that were exposed to E2 showed the development of markedly crowded glands with complex architecture, lined by cells with enlarged, atypical nuclei exhibiting a loss of polarity and variably conspicuous nucleoli, characteristic of complex atypical endometrial hyperplasia (CAEH) (Fig. 7b middle). The endometrium of mice treated with GC-P4_60μg_ following E2-based establishment of non-atypical SEH contained crowded, cystically dilated glands lined by cytologically unremarkable proliferating glandular cells, all characteristic for SEH (Fig. 7b bottom). Tissue histology identified clear differences in between GC-P4_60μg_ treated and non-treated hyperplastic endometrium

## Discussion

The goal of this study is to improve and optimize vaginal P4 delivery by designing a new thermosensitive, mucoadhesive gel using GC, a chitosan derivative, as the delivery platform. The results demonstrate that GC-P4 is a promising P4-delivery system suitable for vaginal application. GC-P4 gel is stable in a vaginal like environment, potentially safe for vaginal tissues, and demonstrates efficacy against the development of CAEH.

Although various hydrogels, represented by copolymers of poly-(ethylene oxide), poly-(propylene oxide), and copolymer of *N*-isopropylacrylamide show promising properties for biomedical applications, their clinical use has been limited by their lack of biodegradability, biocompatibility, and physical stability (31,32). Therefore, there has been a continuing effort to develop new bio-functional polymers with improved biocompatibility and physico-chemical properties. Previously, we have reported a novel thermo-reversible sol–gel transition polymer, glycol chitin (GC), which is synthesized by a simple *N*-acetylation reaction of glycol chitosan (14). In contrast to typical sol–gel transition polymers structurally based on synthetic block copolymer structures, GC is an amphiphilic chitosan-based polymer with good biocompatibility and biodegradability. It is one of few polysaccharide-based polymers autonomously showing a negative thermosensitive sol–gel transition around body temperature without involvement of any crosslinking, grafting or blending systems. In this work, we report for the first time the utilization of GC thermosesitve polymer as a vaginal drug delivery vehicle for P4. Therefore we, evaluated the behavior of GC and GC-P4 in vaginal like environment as described here

Five percent GC of 90% DA used in this study, exhibited stable gelation near body temperature. Moreover, environmental pH appeared to be another factor influencing GC T_gel_. Since the pKa of the residual amine groups on GC backbone is ~6.84, ionization of these amines take place at lower pH inducing the repulsion between polymer chains (33). Thus, increasing T_gel_. On the other hand, association of polymer chains takes place in a higher pH (~7.2) leading to a reduction in T_gel_. These results indicate the 5% GC at pH 4.2 forms a gel near body temperature and vaginal pH, making it a suitable candidate for vaginal application. 5% GC prepared in pH 4.2 can maintain its physical stability (*i.e.* appearance and transparency) after being stored at 4°C for several months (Figure S8)

We have also confirmed the mucoadhesive ability of GC based using a rheological synergism evaluation. It was reported that free amine groups on chitosan backbones are the main driving force for mucoadhesion (16). However, GC used in this study (≈90% degree of acetylation) only exhibits a fairly low number of free amine groups when compared to chitosan, yet still maintained its advantageous mucoadhesive properties (14). We suggest that thermosensitivity of the hydrogel might be contributed to its mucoadhesion. Thermosensitivity could increase the interaction time between GC and the mucinous, vaginal surface probably providing an enhanced establishment of mucoadhesive forces. Unlike other vaginal formulations, which were occasionally reported to melt and leak at body temperature, GC maintains its physical stability within the vaginal environment (2,12). Thus, reducing undesirable vaginal discharges responsible for vaginal and vulvar irritations, possible yeast super infection, and ensuring accurate dosing (3,34).

It is known that some amphiphilic polymers have the ability to enhance the solubility of hydrophobic drugs by forming micelle-like aggregates in aqueous media with hydrophobic domains favoring the partitioning of hydrophobic agents (14,15). Despite the significant enhancement of the aqueous solubility of P4 by GC, the enhancement was still in the range of micrograms and similar to other current methods used to enhance P4 solubility such as using PEG 400: water (1:1) mixture. Since the therapeutic effects of P4 are seen in the milligram dose range, we attempted to increase the amount of loaded P4 in our system (29,35). Several alternative methods have been used to enhance the loaded amount of hydrophobic agents into thermosensitive gels, including dispersing the desired drug to a concentration that exceeds its solubility (9,36). It was reported that saturating extramicellar channels in polymeric hydrogels with drug particles favors the controlled release of such drug without significantly altering T_gel_ (36). Therefore, we developed a dispersion system of 0.1%; (w/v) P4 into 5% GC at pH 4.2 (GC-P4), which maintained an acceptable T_gel_ for mucosal application and thermosensitive properties of the hydrogel (10).

The GC-P4 system described herein has shown several favorable rheological characteristics such as stability and spreadability in vaginal like environment. Here, we showed that GC-P4 microstructure is more resistant to external stress and less prone to liquefy under physiological conditions compared to Crinone®, a commercially available P4 gel. In addition, the preferred shear thinning, non-Newtonian behavior that GC-P4 retained under increasing shear indicates a satisfactory distribution across vaginal walls important for an equal coating for most of the vaginal surface (24). Besides sheering, there are several other factors in the vagina that can contribute to gel flow behavior and distribution. For example, the presence of VFS and SFS can lead to a change in gel-viscosity through diluting the formulation. Despite the dilution of GC-P4 by VFS, the hydrogel still maintains suitable viscosities. However, it seems that the viscosity of GC-P4 drastically decreases in the presence of SFS. It was reported that viscosities of chitosan-based gels decrease by decreasing the final polymer concentration, due to reducing intermolecular entanglements between polymer chains and increasing their free movements (37). Since the dilution of GC-P4 with SFS was in 1:1 ratio resembling physiological volume of semen, we expected to see a significant reduction in viscosity and gel quality compared to dilution with VFS. Therefore, we suggest that the application of GC-P4 is not suitable in the presence of semen such as immediately prior or shortly after vaginal intercourse due to excessive dilution and possible leaking of the formulation.

Stability and durability of a formulation in the vagina are also crucial allowing an effective release time for the loaded active ingredients (30). After exposure to VFS, GC-P4 showed gradual reduction in gel weight due to polymer chains dissolution (erosion) following initial stage of polymer chain relaxation (swelling). These finding suggest that drug release may be governed by P4 diffusion out of the gel coupled with GC erosion, typically seen with release mechanisms of drug from hydrogels (38). The physical presence of GC-P4 hydrogel *in vitro* even after 4 h post exposure to VFS might suggest an extended residence time, due to both its mucoadhesive and thermosensitive properties. Extended residence times can play a crucial role in reducing the frequency of administration required for an effective treatment, and crucial factor to favorably enhancing patient compliance (9). In this case, GC-P4’s provided a controlled-release for P4 liberating up to 50% of the loaded drug within the first four hours post exposure to vaginal like conditions. Controlled-release systems usually require less amounts of drug to achieve the desired therapeutic effect, possibly reducing the cost of therapy (39). We expect that as the erosion process of GC-P4 hydrogel continues over time, more drug will be liberated in a controlled manner providing an effective absorption and a steady P4 tissue concentration, until a phase of rapid release of drug due to the erosion of the final layer of polymeric gel. Although the percentage of cumulative P4 released matches the total percentage of loss in GC-P4 weight, we wanted to exclude the possibility of P4 inactivation either by the GC system or other factors. PR-reporter assays show that P4 successfully maintains its bioactivity following its released from GC-P4, which implies that GC provides a compatible medium for P4 without hampering its bioactivity. These results demonstrate that GC-P4 is indeed a stable hydrogel capable to release biologically active P4 into the vaginal environment.

Another prerequisite for an effective and safe vaginal formulation is its ability to not interfere with the vaginal mucosal integrity and the vaginal ecology (40). Vaginal formulations that are either toxic or non-compatible with the vaginal epithelium can cause local irritation and are associated with increased inflammation and infection rates, complications and hence treatment costs (2,13). We showed that GC is safe to vaginal epithelial tissue and does not cause any apparent disturbance to the vaginal flora. Higher degrees of acetylation may be contributing to GC’s superior safety profile respective to vaginal tissues. Although chitosan is not known to exhibit increased tissue toxicity, it still may hold potential toxicity based on its amount of cationic surface groups (41). Charge-to-charge interactions between cationic materials and anionic cellular membranes can potentially result in cellular membrane damage (41). Therefore, masking cationic charges by acetylating amine groups may explain the low toxicity of GC. A head to head comparison of GC-P4 with the Crinone®, a commercially available vaginal P4 formulation, using Clonogenic assay revealed a significant decrease in viable cell colonies after exposure to Crinone®. This may be due to some of Crinone’s® ingredients, such as sorbic acid known to cause tissue irritation (42). Whereas only a slight impact on colony numbers could be observed after GC-P4 exposure, implying the relative safety of GC-P4 to vaginal mucosa. Similar results were found with bacterial growth inhibition assay, proving the safety of 5% GC and GC-P4 to vaginal flora.

The *in vitro* safety and biocompatibility of GC-P4 to the vaginal environment was further confirmed *in vivo* using a Female Nu/Nu (6–8 week old) mouse model. Mouse models are being commonly used as a suitable and economical alternative for the preclinical assessment of safety and efficacy of novel vaginal formulations (43). In direct comparison to Gynol II®, which exhibited significant toxicity to the mouse vaginal epithelium *in vivo* presented by signs of chronic irreversible inflammation, massive cell death of squamous cells, and neutrophil infiltration, histopathological evaluation of the mouse vaginal epithelium after the application of GC-P4 showed almost no signs of toxicity with only minimal and reversible inflammation spontaneously resolving (44,45). With a favorable outcome of first *in vivo* studies confirming the safety of GC-P4 including multiple applications, additional testing for signs of chronic toxicity needs to be confirmed in future studies. To assess GC-P4’s efficacy *in vivo*, an E2 induced EH mouse model was used. Mice exposed to continuous doses of E2 developed SEH after around 4 weeks, which then after two additional weeks will advance to complex atypical EH (CAEH). No disease progression to CAEH was observed to occur after 2 weeks of GC-P4 treatment. This finding was confirmed based on histo-pathological tissue analyses affirming only signs of SEH without any forms of atypia. On the other hand, untreated mice indeed progressed from SEH to CAEH and some cases even advanced all the way to borderline EC. Therefore, we suggest that P4 released from GC-P4 was able to minimize the proliferative action of E2 on the murine endometrium preventing the progression of SEH to CAEH or even EC. With the initial very promising efficacy, additional *in vivo* animal dose and frequency-finding studies are still required.

## Conclusion

The aim of this work was to improve and optimize the vaginal delivery of P4 by designing a new bio-functional hydrogel formulation equipped with thermosensitive and mucoadhesive characteristics empowering this new formulation to successfully overcome limitations of currently available vaginal P4 dosage forms. GC-P4 hydrogel; 5% GC at pH 4.2 loaded with 0.1% P4, showed a desired sol–gel transition near body temperature forming and maintaining a stable, gel with desirable flow behavior in the challenging vaginal environment. Although viscosity was found to decrease after dilution with certain biological fluids, GC-P4 gel was still able to maintain a satisfactory gel status after exposure to VFS. However, GC-P4 gel administration immediately prior or after vaginal intercourse may not be recommended due to possibly impacting GC-P4’s performance and efficacy. GC-P4 gel is capable of prolonged residence in and exhibits an extended, continued and controlled release of P4. This new formulation has proven to be safe for application to the vaginal epithelium *in vitro* as well as *in vivo* without causing tissue toxicity or irritation even after repeated application. Furthermore, initial *in vivo* efficacy studies in an EH mouse model revealed the ability of GC-P4 to prevent the progression of EH from SEH to CAEH. These results therefore indicate that the GC-P4 formulation is a promising alternative to current vaginal P4 formulations

## Electronic supplementary material

Below is the link to the electronic supplementary material.

ESM 1 (DOCX 4196 kb)

## ABBREVIATIONS

CAEH: Complex atypical endometrial hyperplasia
CFU: Colony forming unit
COS: Critical oscillatory stress
DA: Degree of acetylation
E2: Estrogen
EH: Endometrial hyperplasia
G′: Elastic modulus
G″: Viscous modulus
GC: Glycol chitin
GC-P4: 5% glycol chitin- 0.1% progesterone at pH 4.2
H&E: Hematoxylin and Eosin staining
HRT: Hormonal replacement therapy
IM: Intramuscular
LVR: Linear viscoelastic region
OD: Optical density
P4: Progesterone
s.c.: Subcutaneous
SEH: Simple endometrial hyperplasia
SFS: Seminal fluid simulant
T_gel_: Gelation temperature
VFS: Vaginal fluid simulant
ΔG: Absolute synergism parameter
