# Supplementary material for: Thermosensitive Progesterone Hydrogel: A Safe and Effective New Formulation for Vaginal Application
Source: Pharm Res. 2015 Jan 22;32(7):2266–79. doi: 10.1007/s11095-014-1616-8 (PMC4452141; doi:10.1007/s11095-014-1616-8)
Supplement: Supplementary file 1 — (DOCX 4196 kb) [file 11095_2014_1616_MOESM1_ESM.docx]

Supporting Information

Title: Thermosensitive Progesterone Hydrogel: A Safe and Effective New Formulation for Vaginal Application

Aliyah Almomen, Sungpil Cho, Chieh-Hsiang Yang, Zhenghzeng Li, Elke A. Jarboe, C. Matthew Peterson, Kang Moo Huh*, and Margit M. Janát-Amsbury*

**List of Contents:**

Synthesis and characterization of GC

S1. ^1^H-NMR spectrum of glycol chitin with 90% DA.

S2. Representative temperatures sweep analyses for different 80% DA GC concentrations: 3, 4, 5, 6, 8, and 10 % (w/v) GC, at pH 4.2.

S3. Representative temperatures sweep analyses for different 90% DA GC concentration: 5, 6, and 7 % (w/v) GC, at pH 4.2.

S4. Temperatures sweep analyses for 5% (w/v) GC at different pH: 4.2, 6, and 7.2.

S5. Time sweep analysis indicating GC-P4 maintain a gel state (solid state) even after 12 hours of the gel exposure to 37 °C, and 1:4 dilution with VFS. a) G’ > G” at all time point, and b) tan δ (G” /G’) < 1.

S6. Representative image of the bacterial growth inhibition assay using 5% GC, GC-P4, Crinone**®**, and Gynol II**®.** The black dots in each quarter indicate the position of material placement.

S7. Representative image of the clonogenic assay of VK2/E6E7 cells using 5% GC, GC-P4, Crinone**®**, and Gynol II**®**. and P4. Quantification of colonies with Image J colony counting software (size range of colonies: 2 ~ 200 μm).

S8. 5% GC prepared in pH 4.2 still maintain a stable physical appearance even after 6 months of storage in 4 °C. (Right) freshly prepared 5% GC pH 4.2, (Left) 5% GC pH 4.2 prepared 6 months ago. No apparent difference in physical appearance or transparency

**Synthesis and characterization of GC**

Glycol chitin (0.2 g) was dissolved in 25 mL distilled water and then diluted by 25 mL methanol. Acetic anhydride (acetic anhydride/NH_2_ = 10/1, by molar ratio) was added into the glycol chitosan solution under magnetic stirring. After 24 hr reaction under stirring at room temperature, the polymer was precipitated in acetone, followed by centrifugation and dialysis against distilled water for 3 days using a dialysis membrane (MWCO= 2kDa). Glycol chitin was obtained by lyophilization in a powdered form. Glycol chitin was characterized by ^1^H NMR spectroscopy using a JNM-AL400 spectrometer (Jeol Ltd., Akishima, Japan) operating at 400 MHz. Sample was prepared by dissolving glycol chitin in D_2_O (1.0 wt %). The degree of acetylation (DA) was calculated to 90 % from ^1^H NMR spectra by comparing the integrated signal area of the protons of the glucopyranosyl ring (δ=3.55) with that of methyl protons of acetyl group (δ=1.89).


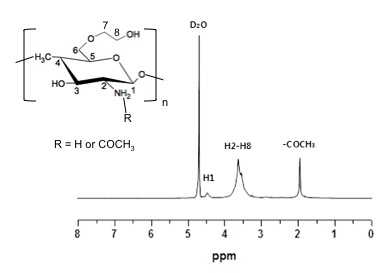


**Figure S1**. ^1^H-NMR spectrum of glycol chitin with 90% DA

**Figure S2**. Representative temperatures sweep analyses for different 80% DA GC concentration: 3, 4, 5, 6, 8, and 10% (w/v) GC, at pH 4.2.


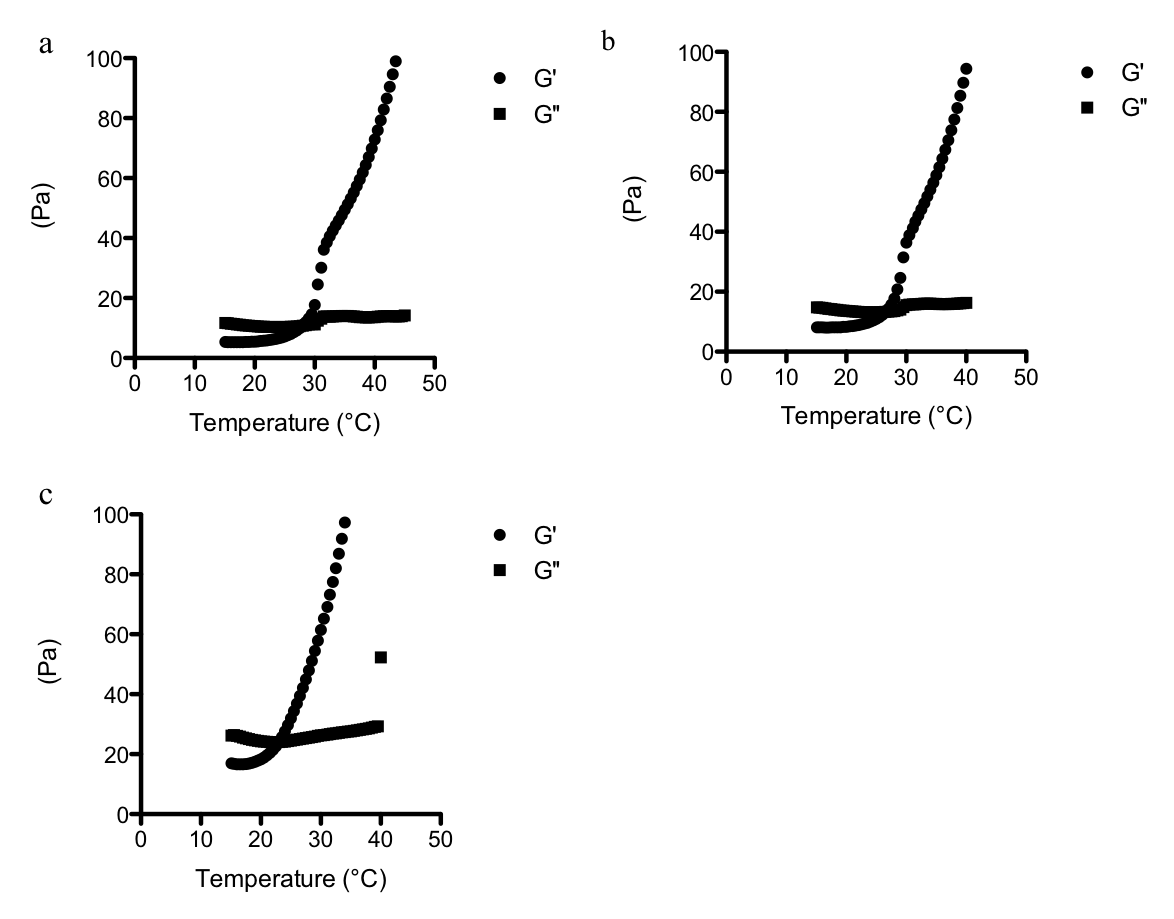


**Figure S3.** Representative temperatures sweep analyses for different 90% DA GC concentration: a) 5, b) 6, and c) 7 % (w/v) GC, at pH 4.2**.**


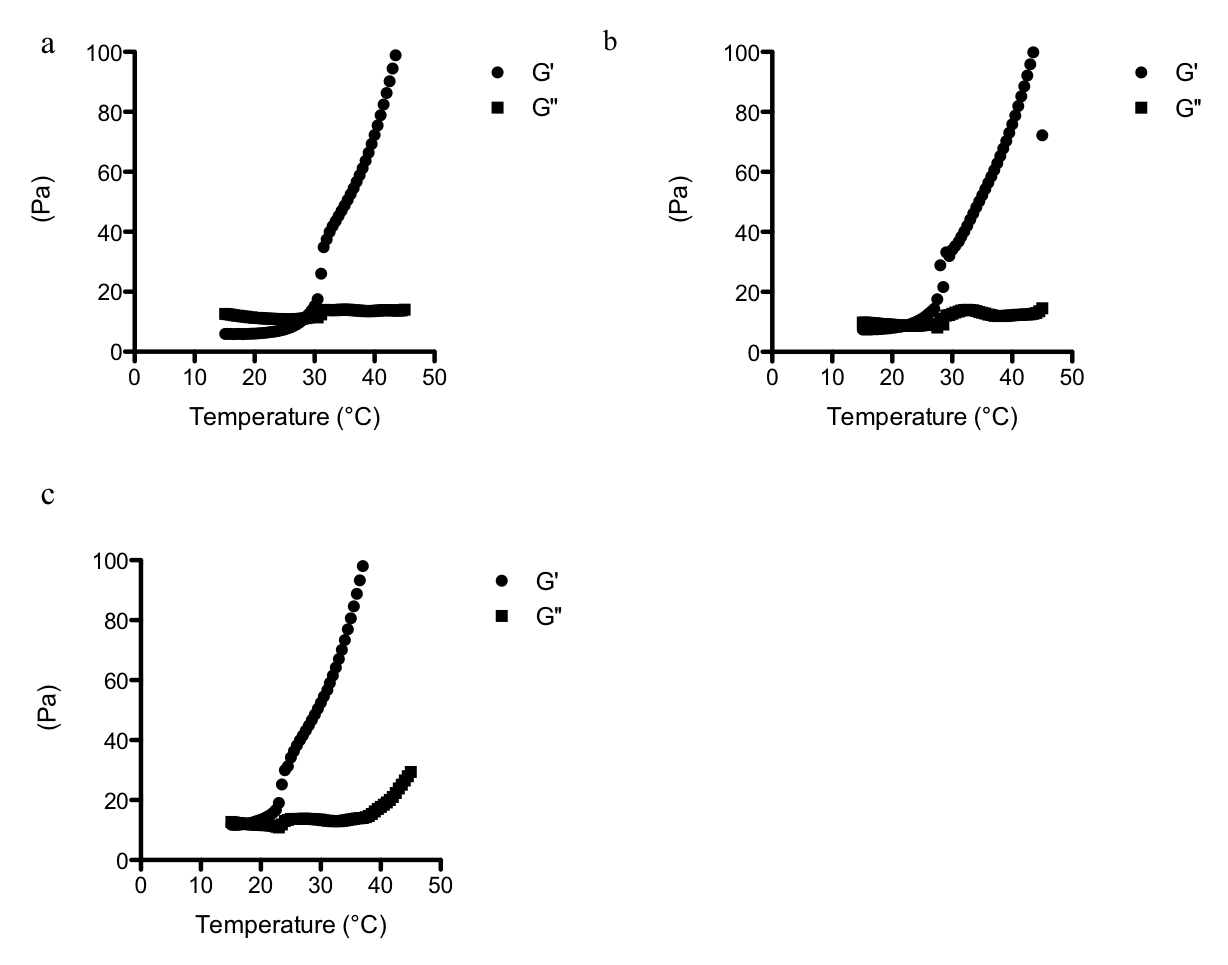


**Figure S4**. Representative Temperatures sweep analyses for 5% (w/v) GC at different pH: a) 4.2, b) 6, and c) 7.2.

a

b

**Figure S5**. Time sweep analysis indicating GC-P4 maintain a gel state (solid state) even after 12 hours of the gel exposure to 37 °C, and 1:4 dilution with VFS. a) G’ > G” at all time point, and b) tan δ (G” /G’) < 1.


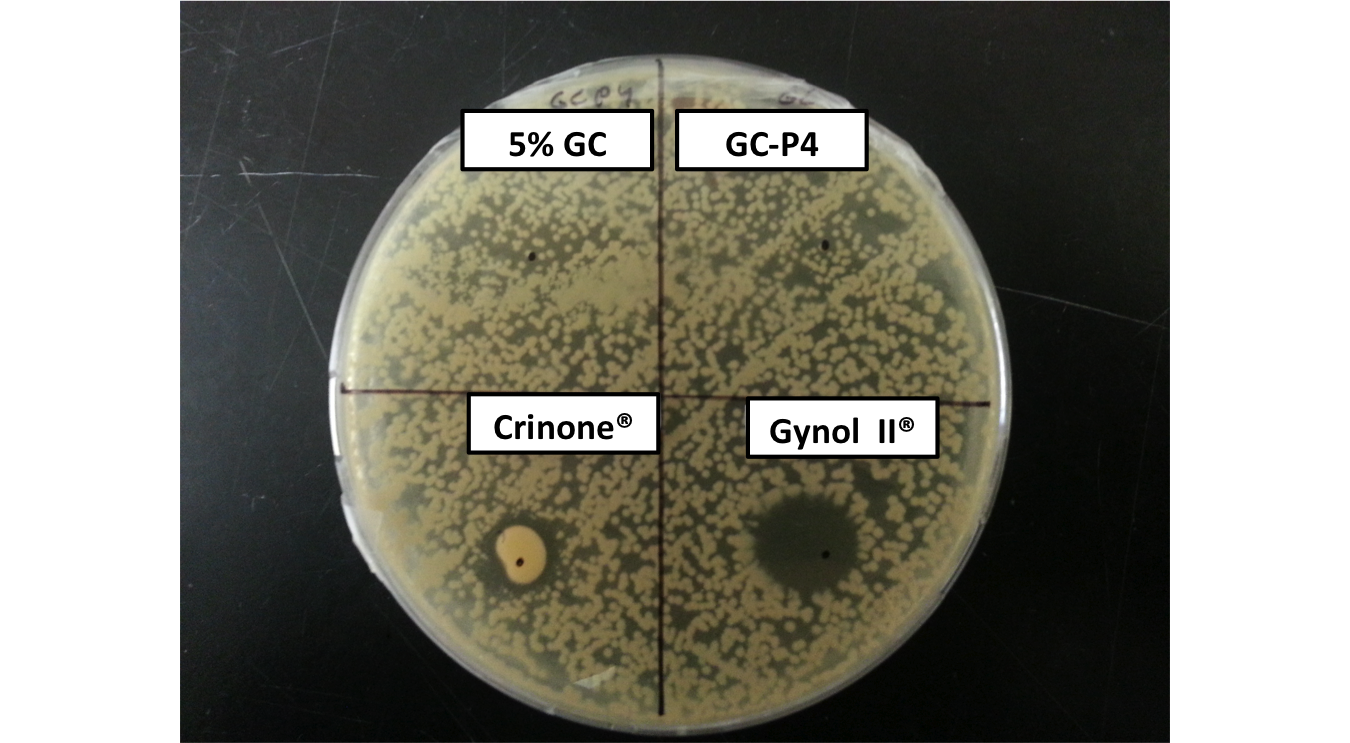


**Figure S6.** Representative image of the bacterial growth inhibition assay using 5% GC, GC-P4, Crinone**®**, and Gynol II**®.** The black dots in each quarter indicate the position of material placement.


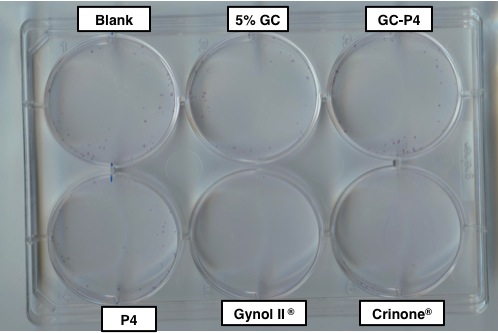


**Figure S7.** Representative image of the clonogenic assay of VK2/E6E7 cells using 5% GC, GC-P4, Crinone**®**, and Gynol II**®**, and P4. Quantification of colonies with Image J colony counting software (size range of colonies: 2 ~ 200 μm).

**
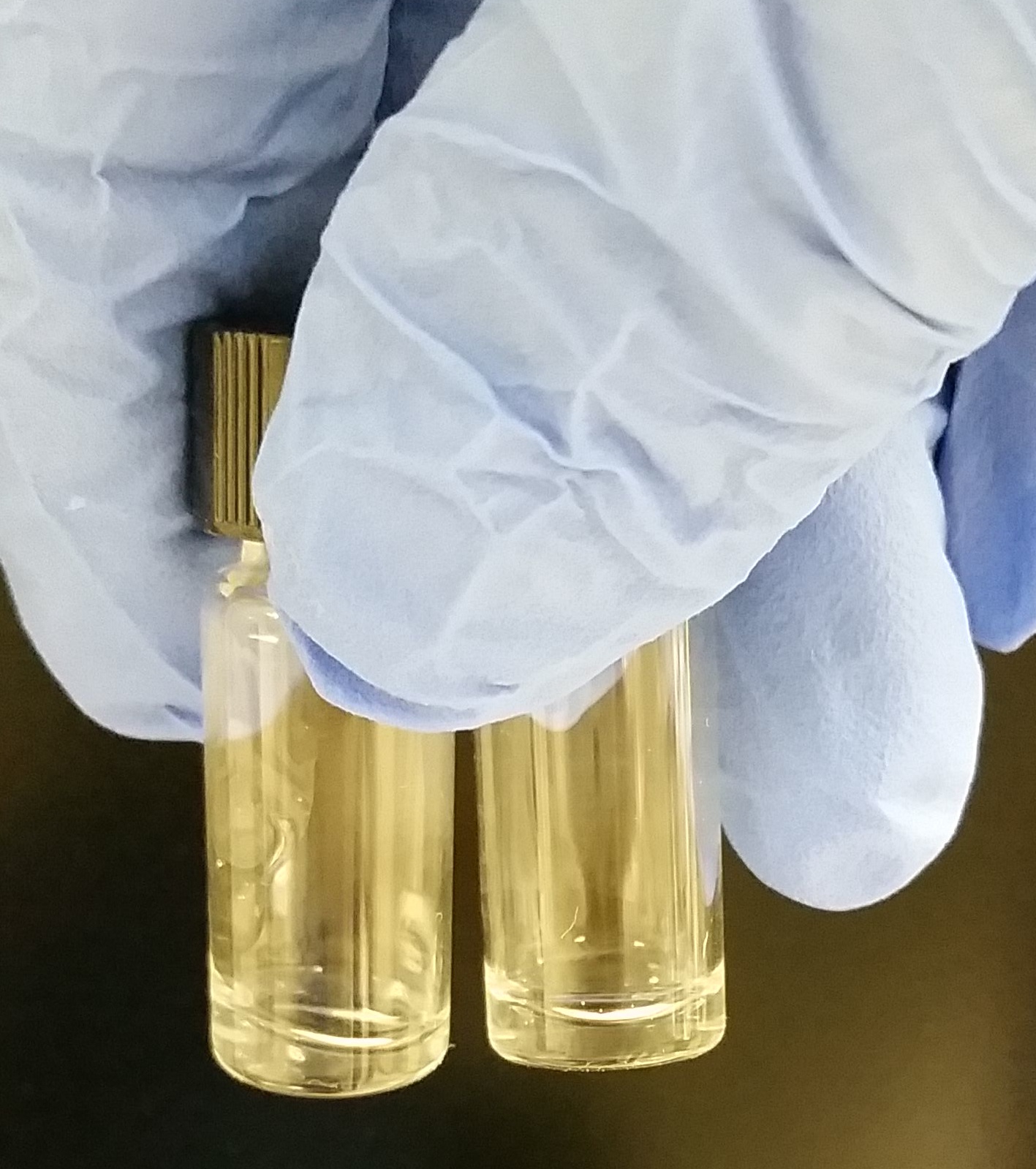
**

Figure S8. 5% GC prepared in pH 4.2 still maintain a stable physical appearance even after 6 months of storage in 4 °C. (Right) freshly prepared 5% GC pH 4.2, (Left) 5% GC pH 4.2 prepared 6 months ago. No apparent difference in physical appearance or transparency
